# Circulating Proprotein Convertase Subtilisin/Kexin Type 9 Levels Predict Future Cardiovascular Event Risks in Hemodialyzed Black African Patients

**DOI:** 10.5041/RMMJ.10443

**Published:** 2021-07-20

**Authors:** François-Pantaléon Musungayi Kajingulu, François Bompeka Lepira, Aliocha Natuhoyila Nkodila, Jean-Robert Rissassy Makulo, Vieux Momeme Mokoli, Pepe Mfutu Ekulu, Justine Busanga Bukabau, Yannick Mayamba Nlandu, Augustin Luzayadio Longo, Nazaire Mangani Nseka, Ernest Kiswaya Sumaili

**Affiliations:** 1 Department of Internal Medicine, Division of Nephrology–Dialysis, University of Kinshasa Hospital, Kinshasa, Democratic Republic of the Congo; 2 Faculty of Family Medicine and Primary Care, Protestant University of Congo, Kinshasa, Democratic Republic of the Congo; 3 Department of Pediatrics, Division of Nephrology–Dialysis, University of Kinshasa Hospital, Kinshasa, Democratic Republic of the Congo

**Keywords:** Black Africans, cardiovascular events, hemodialysis, kidney disease, proprotein convertase subtilisin, kexin type 9

## Abstract

**Context and Objective:**

Cardiovascular diseases are the leading cause of mortality in patients. In this context, proprotein convertase subtilisin/kexin type 9 (PCSK9) appears to be the new biomarker identified as interfering in lipid homeostasis. This study aimed to investigate the association between PCSK9, dyslipidemia, and future risk of cardiovascular events in a population of black Africans.

**Methods:**

A cross-sectional study was conducted between August 2016 and July 2020 in six hemodialysis centers in the city of Kinshasa, Democratic Republic of the Congo. Serum PCSK9 was measured by ELISA; lipid levels of 251 chronic kidney disease grade 5 (CKD G5) hemodialysis patients and the Framingham predictive instrument were used for predicting cardiac events.

**Results:**

Total cholesterol (TC), low-density lipoprotein cholesterol (LDL-c), and triglycerides (TG) were significantly increased in the tertile with the highest PCSK9. By contrast, high-density lipoprotein cholesterol (HDL-c) was significantly decreased in the same tertile. A strong positive and significant correlation was found between PCSK9 and TC, TG, and LDL-c. Negative and significant correlation was observed between PCSK9 and HDL-c. The levels of PCSK9, smoking, overweight, and atherogenic dyslipidemia were associated with future risks for cardiovascular events in univariate analysis. After adjustment, all these variables persisted as independent determinants of future risk for cardiovascular events. The probability of having a cardiovascular event in this population was independently associated with PCSK9 levels. Compared to the patients in the lowest PCSK9 tertile, patients with PCSK9 levels in the middle (aOR 5.9, 95% CI 2.06–17.3, *P*<0.001) and highest tertiles (aOR 8.9, 95% CI 3.02–25.08, *P*<0.001) presented a greater risk of cardiac event.

**Conclusion:**

Increased PCSK9 serum levels are associated with higher levels of TC, LDL-c, and TG and lower levels of HDL-c in black African hemodialysis patients. Serum PCSK9 levels in these patients predict increased risk of cardiovascular events, independent of traditional potential confounders.

## INTRODUCTION

In chronic kidney disease (CKD), patients die more likely from cardiovascular complications than from progression to kidney failure with replacement therapy. Indeed, clinical evidence shows that cardiovascular disease begins as soon as kidney function declines, and its severity increases with the progression of CKD.^1^ However, the cardiovascular risk remains high even in patients undergoing hemodialysis or peritoneal dialysis.^2^,^3^

Among the modifiable cardiovascular risk factors identified, dyslipidemia remains one of the major risk factors in chronic kidney disease grade 5 (CKD G5) hemodialysis patients or CKD without kidney failure with replacement therapy.^4^ Indeed, it has been reported in the literature that the high cardiovascular risk associated with CKD and kidney replacement therapy could be partially explained by the acceleration of the dynamic process of atherosclerosis induced by dyslipidemia.^5^–^7^ In this regard, serum protein proprotein convertase subtilisin/kexin type 9 (PCSK9) has been identified as the main factor inducing the increased synthesis of very-low-density lipoprotein cholesterol (triglyceride-rich lipoprotein), a factor underlying the development of atherogenic dyslipidemia and the subsequent elevated cardiovascular risk.^8^ The plasma protein PCSK9 acts, in large part, by targeting and reducing the hepatic degradation of very-low-density lipoprotein, a lipoprotein containing apolipoprotein B (apo-B) and low-density lipoprotein cholesterol (LDL-c).^9^,^10^

Dyslipidemia (particularly atherogenic dyslipidemia) does not respond well to statins, which partly explains the residual cardiovascular risk observed in patients on statins.^8^,^9^ Several studies report that inhibitors of the plasma protein PCSK9 substantially reduce the residual cardiovascular risk when combined with statins.^8^,^10^–^12^ However, almost all of the studies on the relationship between PCSK9 and cardiovascular disease are conducted in developed countries. Considering the geographic and racial disparity in the prevalence and relative burden of cardiovascular risk factors, it is appropriate for low- and middle-income countries to determine this relationship from a perspective of prevention, screening, and early management of cardiovascular diseases in black African patients. Little is known about PCSK9 levels in CKD,^13^–^15^ and in the single study on this topic, PCSK9 plasma concentration does not predict cardiovascular events in CKD stage 2–4 patients.^16^ Therefore, the present study aimed to investigate the association between PCSK9, dyslipidemia, and future risks of cardiovascular events in a population of black Africans, particularly among patients on chronic kidney disease hemodialysis.

## PATIENTS AND METHODS

### Study Population and Design

In a cross-sectional study, consecutive CKD G5 hemodialysis patients aged >16 years were examined between August 2016 and July 2020 in the following six hemodialysis centers in the city of Kinshasa, the Democratic Republic of Congo: University Hospital of Kinshasa; General Provincial Referral Hospital of Kinshasa; General Referral Hospital of the Congolese National Police; Ngaliema Medical Center Clinic; HJ Hospitals; and AFIA Medical Center.

### Data Collection and Procedure

Variables of interests included: age, sex, CKD etiology, diabetes mellitus (DM), high blood pressure (HBP), smoking, alcoholism, physical inactivity, duration on hemodialysis, and current pharmacological treatment. The physical examination was performed before the hemodialysis session and focused on the following parameters: weight (kg), height (cm), systolic blood pressure (SBP) (mmHg), diastolic blood pressure (DBP) (mmHg), mean arterial pressure (MAP) (mmHg), waist circumference (cm), and pulse and heart rate (beat/min). Laboratory parameters of interest in this study encompassed: serum hemoglobin, hematocrit, urea, creatinine, glycemia, uric acid, total cholesterol (TC), low-density lipoprotein cholesterol (LDL-c), high-density lipoprotein cholesterol (HDL-c), triglycerides (TG), non-HDL-c, calcium, phosphorus, parathyroid hormone (PTHi), vitamin D, and PCSK9. The non-biological parameters included: ankle–brachial pressure index (ABI) (obtained on the basis of the ratio of the systolic pressure of the ankle and that of the brachial) and the body composition of the study population (including: sex, age, height, weight, body fat, muscle mass, and body mass index [BMI]).

The PCSK9 concentration was measured by competitive immunoenzymatic inhibition in accordance with the sandwich enzyme-linked immunosorbent assay (ELISA) human proprotein convertase subtilisin/kexin type 9, MBS920252 Kit (MyBioSource, San Diego, CA, USA). Protein concentrations in the range 0.45 ng/mL to 30 ng/mL were detected. In this study, PCSK9 levels were divided into tertiles: tertile 1: PCSK9 <9.56 ng/L; tertile 2: PCSK9 9.56–23.1 ng/L; and tertile 3: PCSK9 >23.1 ng/L. The lipid fractions were assayed according to the enzymatic colorimetric method on the Cobas C 311, Roche-Paris/France revised version 2010 automated system. Isolated dyslipidemia was defined by a TC ≥200 mg/dL; HDL-c <50 mg/dL in women and <40 mg/dL in men; LDL-c ≥100 mg/dL; or TG ≥150 mg/dL.^17^ Combined dyslipidemia was defined according to the international Frederickson classification: type I or IV dyslipidemia corresponded to a level of LDL-c <100 mg/dL and TG ≥150 mg/dL; type IIa: LDL-c ≥100 mg/dL and TG <150 mg/dL; type IIb: LDL-c ≥100 mg/dL and TG ≥150 mg/dL.^18^ We estimated the risk of cardiovascular events using the Framingham equation adopted by the World Health Organization (WHO) and the *Agence nationale d’accréditation et d’évaluation en santé* (ANAES) in 2004, which made it possible to predict the 10-year risk of a major cardiovascular event (myocardial infarction, cerebrovascular accident, angina, or sudden death). This model takes into account the following parameters: sex, age, systolic blood pressure, TC, HDL-c, smoking, antihypertensive treatment, diabetes mellitus, and family history of hypertension. Framingham scores of <20%, 20%–30%, and >30% were considered as low risk, high risk, and very high risk, respectively.

Written informed consent was obtained from all participants before enrollment. This study protocol was submitted to the ethics committee of the School of Public Health of Kinshasa/DRC for analysis and received approval registered at number: ESP/CE/053/2016.

### Statistical Analysis

All data were analyzed using SPSS for Windows version 24 software. The descriptive statistics were: the mean and standard deviation for quantitative variables with Gaussian distribution; the median and interquartile range (IQR) for variables not normally distributed; and the relative (%) and absolute (*n*) proportions for categorical data. Student’s *t* test was performed to compare two means. The ANOVA test was used for multiple comparisons. ANOVA tests which were significant at the *P*<0.05 level were supplemented by a post hoc Scheffé test. For the comparison of the medians of two groups, we used the Mann–Whitney *U* test, and for more than two medians the Kruskal–Wallis *H* test. The linear regression test was applied to check the correlation between the different lipid fractions and PCSK9. Pearson’s correlation coefficients (*r*) were calculated to assess this association. The search for the determinants of high future risks of cardiovascular event was carried out using the multivariate binary logistic regression model. The adjusted ORs and their 95% CIs were calculated to determine the degree of association between elevated future cardiovascular risks and the independent variables. The significance level retained was *P* value <0.05.

## RESULTS

**Figure 1:**
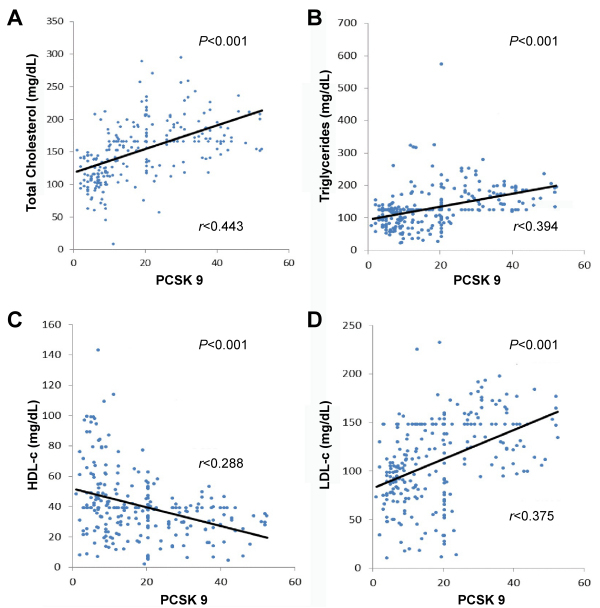
Correlation Between PCSK9 and Lipid Profile in the Study Population. **A:** Correlation between PCSK9 and total cholesterol. **B:** Correlation between PCSK9 and triglycerides. **C:** Correlation between PCSK9 and HDL-c. **D:** Correlation between PCSK9 and LDL-c.

In total, 251 adult CKD G5 hemodialysis patients were enrolled: 182 (72.5%) men and 69 (27.5%) women. Their average age was 55.8±13.6 years (Table 1). The main cardiovascular risk factors encountered in this population were hypertension (87.3%), diabetes mellitus (28.3%), alcohol consumption (47.0%), smoking (20.3%), and gout (7.2%). The distribution of lipid particles studied was: low HDL-c 74.5%; high LDL-c 33.1%; high TG 29.1%; and high TC 15.1%. Atherogenic dyslipidemia, abnormal ABI, and high cardiovascular risk were observed in 77.3%, 24.3%, and 25.9%, respectively. Considering the clinical and biological characteristics (Table 2), TC, LDL-c, and TG increase significantly from tertile 1 to tertile 3, while HDL-c decreases significantly across the same tertiles. A strong positive and significant correlation was found between PCSK9 and TC, TG, and LDL-c (Figure 1). On the other hand, a negative and significant correlation was shown between PCSK9 and HDL-c.

**Table 1:** Description of the Study Population Characteristics.

Variables	Overall *n*=251	Tertile 1 *n*=84	Tertile 2 *n*=83	Tertile 3 *n*=84	*P* Value
Age	55.8±13.6	55.1±13.4	57.6±14.9	54.7±12.3	0.338

Sex					0.293
Male	182 (72.5)	66 (78.6)	57 (68.7)	59 (70.2)	
Female	69 (27.5)	18 (21.4)	26 (31.3)	25 (29.8)	

HBP	219 (87.3)	73 (86.9)	77 (92.8)	69 (82.1)	0.149

DM	71 (28.3)	22 (26.2)	22 (26.5)	27 (32.1)	0.754

Gout	18 (7.2)	7 (8.3)	3 (3.6)	8 (9.5)	0.440

CVD	58 (23.1)	19 (22.6)	21 (25.3)	18 (21.4)	0.719

Physical Inactivity	233 (92.8)	78 (92.9)	78 (94.0)	77 (91.7)	0.858

Smoking	51 (20.3)	17 (20.2)	9 (10.8)	25 (29.8)	0.010

Alcohol	118 (47.0)	35 (41.7)	42 (50.6)	41 (48.8)	0.480

NSAIs	47 (18.7)	16 (19.0)	19 (22.9)	12 (14.3)	0.373

Obesity	22 (8.8)	5 (6.0)	8 (9.6)	9 (10.7)	0.570

Overweight	58 (23.1)	25 (29.8)	19 (22.9)	14 (16.7)	0.146

HCV Ac	11 (5.2)	5 (6.8)	5 (7.1)	1 (1.5)	0.241

HBs Ag	6 (2.9)	1 (1.4)	3 (4.3)	2 (3.0)	0.609

HIV Ac	6 (2.9)	2 (2.7)	1 (1.4)	3 (4.5)	0.529

TC High	38 (15.1)	2 (2.4)	14 (16.9)	22 (26.2)	<0.001

HDL-c Low	187 (74.5)	47 (56.0)	64 (77.1)	76 (90.5)	<0.001

LDL-c High	83 (33.1)	8 (9.5)	25 (30.1)	50 (59.5)	<0.001

TG High	73 (29.1)	4 (4.8)	18 (21.7)	51 (60.7)	<0.001

AD	194 (77.3)	48 (57.1)	64 (77.1)	82 (97.6)	<0.001

ABI	1.16±0.18	1.10±0.11	1.15±0.18	1.22±0.22	<0.001
Normal	190 (75.7)	79 (94.0)	64 (77.1)	47 (56.0)	
Abnormal	61(24.3)	5 (6.0)	19 (22.9)	37 (44.0)	

CVR/10 years					<0.001
Low	186 (74.1)	78 (92.9)	61 (73.5)	47 (56.0)	
High	65 (25.9)	6 (7.1)	22 (26.5)	37 (44.0)	

Data are expressed as: mean±standard deviation; or *n* (%).

ABI, ankle–brachial index; AD, atherogenic dyslipidemia; CVD, cardiovascular diseases; CVR, cardiovascular risk; DM, diabetes mellitus; HBP, high blood pressure; HBs Ag, hepatitis B surface antigen; HCV Ac, hepatitis C virus antibodies; HDL-c, high-density lipoprotein cholesterol; HIV Ac, human immunodeficiency virus antibodies; LDL-c, low-density lipoprotein cholesterol; NSAIs, non-steroidal anti-inflammatory drugs; TC, total cholesterol; TG, triglycerides.

**Table 2:** Clinical and Biological Study Population Characteristics by PCSK9 Tertile.

Variables	Overall *n*=251	Tertile 1 *n*=84	Tertile 2 *n*=83	Tertile 3 *n*=84	*P* Value
SBP (mmHg)	154.8±21.5	155.6±20.3	153.9±21.5	154.8±22.7	0.871
DBP (mmHg)	88.4±13.6	88.3±13.9	88.3±13.5	88.7±13.4	0.978
MAP (mmHg)	110.5±14.4	110.7±14.5	110.2±14.3	110.7±14.5	0.959
PP (mmHg)	66.4±17.7	67.4±16.4	65.6±17.7	66.2±18.9	0.801
RP (ppm)	89.4±13.8	89.7±13.9	88.9±14.4	89.8±13.3	0.917
BMI (kg/m^2^ )	23.5±4.8	23.5±4.3	23.3±4.3	23.8±5.6	0.828
HbA1c (%)	7.1±3.1	5.7±0.5	8.2±4.3	6.5±1.2	0.291
Hb (g/dL)	8.5±1.9	8.2±1.7	8.6±1.9	8.9±2.0	0.048
Creatine (mg/dL)	10.2 (9.6–11.2)	10.6 (9.4–12.0)	9.9 (8.2–10.8)	11.0 (9.4–13.8)	0.629
eGFR (mL/min/1.73 m^2^)	6.0 (5.6–6.69)	6.2 (5.6–7.2)	6.7 (5.8–7.9)	5.4 (4.6–6.9)	0.609
Urea (mg/dL)	146.0 (131.8–163.5)	154.1 (127.9–180)	139.4 (116–153)	155 (125.8–197)	0.064
Uric Acid (mg/dL)	11.7±19.8	7.5±2.9	10.4±2.8	15.5±30.1	0.597
TC (mg/dL)	152.8±48.2	114.4±28.8	165.4±44.7	178.9±43.2	<0.001
HDL-c (mg/dL)	40.3±22.1	52.0±27.4	37.6±18.9	31.1±11.9	<0.001
LDL-c (mg/dL)	110.7±44.1	90.1±34.3	102.5±46.8	139.5±34.3	<0.001
TG (mg/dL)	132.3±63.9	103.5±35.2	125.5±83.1	167.8±45.8	<0.001
tCa (mmol/L)	2.3 (2.1–2.4)	2.5 (2.1–2.8)	1.9 (1.8–2.3)	2.3 (2.0–2.7)	0.489
iCa (mmol/L)	1.0 (0.9–1.03)	1.0 (0.9–1.03)	0.98 (0.89–1.03)	0.99 (0.89–1.03)	0.310
Ph (mmol/L)	2.2 (1.9–2.8)	1.6 (1.3–2.1)	2.0 (1.6–2.9)	2.9 (2.6–2.95)	0.219
PTHi (ng/L)	181.6 (123.5–192.3)	184.4 (88.9–192.3)	192.3 (98.4–192.3)	177.4 (80.7–192.3)	0.281
Vitamin D (ng/L)	52.2 (46.3–64.0)	36.4 (28.7–49.9)	38.5 (28.5–60.5)	65.7 (65.4–65.7)	<0.001
FM (%)	27.0±9.3	27.8±9.1	26.6±9.5	26.6±9.5	0.661
MM (%)	25.7±8.4	24.6±8.3	25.3±7.4	26.9±9.4	0.183

Data are expressed as: mean±standard deviation; or median (IQR).

BMI, body mass index; DBP, diastolic blood pressure; eGFR, estimated glomerular filtration rate; FM, fat mass; Hb, hemoglobin; HbA1c, glycated hemoglobin; HDL-c, high-density lipoprotein cholesterol; iCa, ionized calcium; LDL-c, low-density lipoprotein cholesterol; MAP, mean arterial pressure; MM, muscular mass; Ph, phosphorus; PP, pulse pressure; PTHi, intact parathormone; RP, radial pulse; SBP, systolic blood pressure; TC; total cholesterol; tCa, total calcium; TG, triglycerides.

Table 3 shows that the high cardiovascular risk was observed in patients having higher frequency of alcohol consumption, smoking, overweight, and abnormal ABI. This risk was proportional to the increase in plasma TC, LDL-c, and TG levels. The risk was significantly elevated for low HDL-c levels. Patients of the study with PCSK9 level in tertile 3 also had an elevated cardiovascular risk.

**Table 3:** Population Characteristics by Cardiovascular Risk Using the Framingham Predictive Instrument.

Variables	CVR Low *n*=186	CVR High *n*=65	*P* Value
Sex			0.138
Male	131 (70.4)	51 (78.5)	
Female	55 (29.6)	14 (21.5)	

HBP	161 (86.6)	58 (89.2)	0.757

DM	51 (27.4)	20 (30.8)	0.800

Gout	12 (6.5)	6 (9.2)	0.833

CVD	41 (22.0)	17 (26.2)	0.721

Physical Inactivity	175 (94.1)	58 (89.2)	0.316

Smoking	27 (14.5)	24 (36.9)	<0.001

Alcohol	80 (43.0)	38 (58.5)	0.023

NSAIs	33 (17.7)	14 (21.5)	0.307

Obesity	15 (8.1)	7 (10.8)	0.331

Overweight	36 (19.4)	22 (33.8)	0.015

HCV Ac	9 (5.7)	2 (3.8)	0.444

HBs Ag	3 (1.9)	3 (5.7)	0.170

HIV Ac	5 (3.2)	1 (1.9)	0.527

TC	24 (12.9)	14 (21.5)	0.040

HDL-c Low	126 (67.7)	61 (93.8)	<0.001

LDL-c High	41 (22.0)	42 (64.6)	<0.001

TG	43 (23.1)	30 (46.2)	0.001

AD	132 (71.0)	62 (95.4)	<0.001

ABI			0.010
Normal	145 (78.0)	45 (69.2)	
Abnormal	41 (22.0)	20 (30.8)	

PCSK9			<0.001
Tertile 1	78 (41.9)	6 (9.2)	
Tertile 2	61 (32.8)	22 (33.8)	
Tertile 3	47 (25.3)	37 (56.9)	

Data are expressed as: mean±SD.

ABI, ankle–brachial systolic pressure index; AD, atherogenic dyslipidemia; DM, diabetes mellitus; CVD, cardiovascular disease; CVR, cardiovascular risk; HBs Ag, hepatitis B surface antigen; HCV Ac, hepatitis C virus antibodies; HDL-c, high-density lipoprotein cholesterol; HIV Ac, human immunodeficiency virus antibodies; HBP, high blood pressure; LDL-c, low-density lipoprotein cholesterol; NSAIs, non-steroidal anti-inflammatory drugs; PCSK9, proprotein convertase subtilisin/kexin type 9; TC, total cholesterol; TG, triglycerides.

In a univariate analysis, smoking, overweight, atherogenic dyslipidemia, and PCSK9 levels were associated with future risks of cardiovascular events (Table 4). Following the adjustment, all these variables persisted as independent determinants of future risks for cardiovascular events.

**Table 4:** Determinants of Future Cardiovascular Events in the Study Population.

Variables	*P* Value	Unadjusted OR (95% CI)	*P* Value	Adjusted OR (95% CI)
Smoking				
No		1		1
Yes	<0.0001	3.45 (1.80–6.59)	0.004	3.38 (1.48–7.72)

Alcohol				
No		1		1
Yes	0.033	1.87 (1.05–3.31)	0.441	1.32 (065–2.66)

Overweight				
No		1		1
Yes	0.018	2.13 (1.14–4.00)	0.003	3.17 (1.47–6.81)

AD				
No		1		
Yes	<0.001	8.46 (2.54–28.10)	0.026	4.30 (1.20–15.49)

PCSK9				
Tertile 1		1		1
Tertile 2	0.002	4.69 (1.79–12.28)	0.001	5.98 (2.06–17.37)
Tertile 3	<0.0001	10.23 (4.02–26.08)	<0.0001	8.91 (3.09–25.72)

ABI				
Normal		1		1
Abnormal	0.016	1.57 (1.37–2.95)	0.693	1.16 (0.55–2.47)

ABI, ankle–brachial pressure index; AD, atherogenic dyslipidemia; CI, confidence interval; OR, odds ratio; PCSK9, proprotein convertase subtilisin/kexin type 9.

Indeed, the probability of having a cardiovascular event in this population is strongly associated with the PCSK9 level. Hence, compared to patients in tertile 1, patients with PCSK9 levels in tertile 2 and tertile 3 had a 6- and 9-fold higher OR, respectively.

## DISCUSSION

This study identified, for the first time, to the best of our knowledge, a positive and significant correlation between PCSK9 and TC, LDL-c, and TG in sub-Saharan African hemodialysis patients. In addition, plasma PCSK9 levels are independently associated with future risks of cardiovascular events. The strong positive and significant correlation found in this study between PCSK9 and TC, TG, and LDL-c is similar to that reported elsewhere.^13^–^15^,^19^–^21^ In some studies of non-diabetic obese, this correlation has not been shown.^22^,^23^ This result suggests that this correlation requires certain metabolic conditions. The emerging role of PCSK9 is observed in different conditions of cardiovascular homeostasis focused on dyslipidemia, glomerular proteinuria, insulin secretion, regulation of blood pressure, and inflammation.^24^ Patients included in our study had a high frequency of hypertension, DM, tobacco consumption, and alcohol intake. This correlation found between PCSK9 and the lipid fractions would explain the cardiovascular risk encountered in hemodialysis patients.^25^ Indeed, given that PCSK9’s main site of action is the membrane LDL-c receptor where a growth factor acts as a ligand in the presence of the catalytic domain of PCSK9, this synergy catabolizes the LDL-c receptors. Therefore, the plasma level of LDL-c increases by decreasing the LDL-c receptors. The LDL-c particles containing apo-B exhibit a high concentration of triglycerides which are very sensitive to oxidation. These particles are the basis of the atherogenic process,^19^ the final consequence of which is cardiovascular disease. In addition, PCSK9, associated with the metabolism of triglycerides, has the ability to hydrolyze lipoprotein lipase, an enzyme that breaks down triglycerides into fatty acids. Hydrolysis of lipoprotein lipase is the basis for the increase in plasma triglycerides.^19^ Indeed, in patients without cardiac disease, it has been noted that any increase in the level of PCSK9 implies an increase in the cardiovascular risk.^26^ In our study (Table 4), the probability of having a cardiovascular event in this population is associated with the PCSK9 level. Compared to tertile 1, patients with PCSK9 levels in tertiles 2 and 3 had 6- and 9-fold higher OR, respectively. A regulator of LDL-c receptors, PCSK9 is involved in cholesterol homeostasis. The increase of its level is influenced by the activation of platelets,^27^ vascular stiffness,^28^ and coronary plaque instability.^29^ Therefore, the level of PCSK9 is not a predicting factor of cardiovascular disease in CKD patients at early stages, such as stages 2–3 of CKD,^16^,^30^ but in patients with stages 4–5 of CKD. In our study, the patients included are black African CKD patients undergoing CKD G5 hemodialysis in whom traditional and non-traditional black African CKD patients undergoing CKD G5 hemodialysis in whom traditional and non-traditional cardiovascular risk factors coexist. The reason that the PCSK9 level is associated with dyslipidemia in our study is not elucidated and could be explained by genetic or environmental factors. However, these possibilities and other explanations require continued investigation. Indeed, genetic variants significantly associated with dyslipidemia have already been identified: in a Canadian study, loss of gene function was found in the Caucasian population but not in the population of African origin.^31^,^32^ Considering these observations, our study suggests that the relationship between PCSK9 and dyslipidemia in black Africans undergoing hemodialysis may be due to a genetic predisposition that could be gains-of-function of PCSK9 in these patients. This observation is consistent with the study conducted in Kenya in a black African population that showed that the plasma PCSK9 level was significantly associated with plasma LDL-c level, and a strong association with plasma LDL-c level, and a strong association was observed between PCSK9 and triglycerides.^33^

Several limitations exist in this study, including its cross-sectional design, the relatively small sample size, and the method used to predict risk of future cardiovascular events. Thus, these findings require confirmation in a larger cohort of prospective African samples.

## CONCLUSION

This study suggests that a higher plasma level of PCSK9 is associated with a higher Framingham score. Therefore, PCSK9 could be used as a biomarker for the prediction of cardiovascular events in black African hemodialysis patients. This preliminary study suggests the importance of future studies on the effect of PCSK9 in cardiovascular events as well the worse survival encountered in African hemodialysis patients.

## Abbreviations

ABI: ankle–brachial systolic pressure index
HDL-c: high-density lipoprotein cholesterol
LDL-c: low-density lipoprotein cholesterol
PCSK9: proprotein convertase subtilisin/kexin type 9
TC: total cholesterol
TG: triglycerides
VLDL-c: very-low-density lipoprotein cholesterol

## References

[1] Hermans MP, Ahn SA, Rousseau MF (2012). The atherogenic dyslipidemia ratio [log (TG)/HDL-c] is associated with residual vascular risk, beta-cell function loss and microangiopathy in type 2 diabetes females. Lipids Health Dis.

[2] Chandrashekar A, Ramakrishnan S, Rangarajan D (2014). Survival analysis of patients on maintenance hemodialysis. Indian J Nephrol.

[3] Neves M, Machado S, Rodrigues L (2014). Cardiovascular risk in peritoneal dialysis - a Portuguese multicenter study. Nefrologia.

[4] Wolf G, Ziyadeh FN (1999). Molecular mechanisms of diabetic renal hypertrophy. Kidney International.

[5] Shirai K (2004). Obesity as the core of the metabolic syndrome and the management of coronary heart disease. Curr Med Res Opin.

[6] Suzuki M, Takamisawa I, Suzuki K (2004). Close association of endothelial dysfunction with insulin resistance and carotid wall thickening in hypertension. Am J Hypertens.

[7] Engole YM, Lepira FB, Nlandu YM (2021). Prognostic significance of abnormal ankle–brachial index among long-term hemodialysis patients in Kinshasa, the Democratic Republic of the Congo. Rambam Maimonides Med J.

[8] Rashid S, Kastelein JJP (2013). PCSK9 and resistin at the crossroads of the atherogenic dyslipidemia. Expert Rev Cardiovasc Ther.

[9] Guardiola M, Plana N, Ibarretxe D (2015). Circulating PCSK9 levels are positively correlated with NMR-assessed atherogenic dyslipidaemia in patients with high cardiovascular risk. Clin Sci (Lond).

[10] Tavori H, Rashid S, Fazio S (2015). On the function and homeostasis of PCSK9: reciprocal interaction with LDLR and additional lipid effects. Atherosclerosis.

[11] Berman AN, Blankstein R (2019). Optimizing dyslipidemia management for the prevention of cardiovascular disease: a focus on risk assessment and therapeutic options. Curr Cardiol Rep.

[12] Brandts J, Müller-Wieland D (2019). PCSK9 inhibition: new treatment options and perspectives to lower atherosclerotic lipoprotein particles and cardiovascular risk. Curr Atheroscler Rep.

[13] Konarzewski M, Szolkiewicz M, Sucajtys-Szulc E (2014). Elevated circulating PCSK-9 concentration in renal failure patients is corrected by renal replacement therapy. Am J Nephrol.

[14] Rasmussen LD, Bøttcher M, Ivarsen P (2020). Association between circulating proprotein convertase subtilisin/kexin type 9 levels and prognosis in patients with severe chronic kidney disease. Nephrol Dial Transplant.

[15] Bermudez-Lopez M, Forne C, Amigo N (2019). An in-depth analysis shows a hidden atherogenic lipoprotein profile in non-diabetic chronic kidney disease patients. Expert Opin Ther Targets.

[16] Rogacev KS, Heine GH, Silbernagel G (2016). PCSK9 plasma concentrations are independent of GFR and do not predict cardiovascular events in patients with decreased GFR. PLoS One.

[17] Brunezl JD, Davidson M, Furberg CD (2008). Lipoprotein management in patients with cardiometabolic risk: consensus statement from the American Diabetes Association and the American College of Cardiology Foundation. Diabetes Care.

[18] Quispe R, Hendrani AD, Baradaran-Noveiry B (2019). Characterization of lipoprotein profiles in patients with hypertriglyceridemic Fredrickson-Levy and Lees dyslipidemia phenotypes: the Very Large Database of Lipids Studies 6–7. Arch Med Sci.

[19] Zhu YM, Anderson TJ, Sikdar K (2015). Association of proprotein convertase subtilisin/kexin type 9 (PCSK9) with cardiovascular risk in primary prevention. Arterioscler Thromb Vasc Biol.

[20] Baass A, Dubuc G, Tremblay M (2009). Plasma PCSK9 is associated with age, sex, and multiple metabolic markers in a population-based sample of children and adolescents. Clin Chem.

[21] Cui Q, Ju X, Yang T (2010). Serum PCSK9 is associated with multiple metabolic factors in a large Han Chinese population. Atherosclerosis.

[22] Mayne J, Raymond A, Chaplin A (2007). Plasma PCSK9 levels correlate with cholesterol in men but not in women. Biochem Biophys Res.

[23] Alborn WE, Cao G, Careskey HE (2007). Serum proprotein convertase subtilisin kexin type 9 is correlated directly with serum LDL cholesterol. Clin Chem.

[24] Hachem A, Hariri E, Saoud P, Lteif C, Lteif L, Welty F (2017). The role of proprotein convertase subtilisin/kexin type 9 (PCSK9) in cardiovascular homeostasis: a non-systematic literature review. Curr Cardiol Rev.

[25] Hwang HS, Kim JS, Kim YG (2020). Circulating PCSK9 level and risk of cardiovascular events and death in hemodialysis patients. J Clin Med.

[26] Leander K, Mälarstig A, Van’t Hooft FM (2016). Circulating proprotein convertase subtilisin/kexin type 9 (PCSK9) predicts future risk of cardiovascular events independently of established risk factors. Circulation.

[27] Navarese EP, Kolodziejczak M, Winter MP (2017). Association of PCSK9 with platelet reactivity in patients with acute coronary syndrome treated with prasugrel or ticagrelor: the PCSK9-REACT study. Int J Cardiol.

[28] Ruscica M, Ferri N, Fogacci F (2017). Circulating levels of proprotein convertase subtilisin/kexin type 9 and arterial stiffness in a large population sample: data from the Brisighella heart study. J Am Heart Assoc.

[29] Cheng JM, Oemrawsingh RM, Garcia-Garcia HM (2016). PCSK9 in relation to coronary plaque inflammation: results of the ATHEROREMO-IVUS study. Atherosclerosis.

[30] Balla S, Nusair MB, Alpert MA (2013). Risk factors for atherosclerosis in patients with chronic kidney disease: recognition and management. Curr Opin Pharmacol.

[31] Babiak J, Rudel LL (1987). Lipoproteins and atherosclerosis. Baillieres Clin Endocrinol Metab.

[32] Austin MA, Hutter CM, Zimmern RL, Humphries SE (2004). Genetic causes of monogenic heterozygous familial hypercholesterolemia: a HuGE prevalence review. Am J Epidemiol.

[33] Paquette M, Luna Saavedra YG, Chamberland A (2017). Association between plasma proprotein convertase subtilisin/kexin type 9 and the presence of metabolic syndrome in a predominantly rural-based sub-Saharan African population. Metab Syndr Relat Disord.

